# Hendra virus: research progress on a bat-borne threat lurking at the human-animal interface

**DOI:** 10.3389/fvets.2026.1889103

**Published:** 2026-08-19

**Authors:** Yanqin Duan, Menghan Xu, Xuan Huang, Qi Liu

**Affiliations:** 1 School of Basic Medicine, Dali University, Dali, China; 2 Yunnan Key Laboratory of Screening and Research on Anti-pathogenic Plant Resources from Western Yunnan, Dali, China

**Keywords:** encoded proteins, Hendra virus, membrane fusion, transmission of Hendra virus, vaccines

## Abstract

Hendra virus (HeV) is a highly lethal zoonotic *henipavirus* that has caused recurrent cross-species spillover events in Australia since 1994. Its capacity for cross-species spillover, together with its tropism for vascular endothelial and neural tissues, makes HeV important to veterinary medicine and public health. Flying foxes are the natural reservoir hosts, and horses act as intermediate and amplifying hosts through which all confirmed human infections have occurred. Detection of antibodies and viral RNA from related *henipaviruses* in Southeast Asian bats indicates regional *henipavirus* diversity and a possible broader *henipavirus* spillover risk, but does not demonstrate HeV circulation or transmission in Southeast Asia. Human HeV infection is rare but can cause severe respiratory and neurological disease. From a veterinary-oriented One Health perspective, this review integrates current evidence on viral biology, transmission, clinical disease, diagnostics, animal models, equine vaccination, and clinical-stage vaccines and antibody-based interventions, with particular attention to HeV-g2-associated diagnostic challenges and the role of equine vaccination in reducing occupational exposure.

## 1 Introduction

HeV is a highly lethal, zoonotic, single-stranded negative-sense RNA virus belonging to the genus *Henipavirus* within the family *Paramyxoviridae* (1). It is the type species and the first formally identified member of the genus *Henipavirus*, which belongs to the subfamily *Orthoparamyxovirinae* of the family *Paramyxoviridae* in the order *Mononegavirales* (2). Owing to its similarity to another subsequently reported *paramyxovirus*, Nipah virus (NiV), HeV was assigned to the genus *Henipavirus*, which was named in 2000 by the *International Committee on Taxonomy of Viruses* (ICTV). NiV has caused nearly annual human infections in Bangladesh (3). On 30 January 2026, the World Health Organization issued a Disease Outbreak News report on two laboratory-confirmed NiV infections in West Bengal, India (4). In response, several Asian countries and regions strengthened surveillance and preventive measures. In August 2022, investigators reported Langya virus (LayV), a zoonotic shrew-borne *paramyxovirus* detected in febrile patients in eastern China (5). Reported LayV infections were generally associated with febrile illness and laboratory abnormalities; no deaths were recorded in the initial cohort of 35 patients (6). Currently, other *henipaviruses* such as Cedar virus (CedV) and Camp Hill virus have also been reported, but none have been confirmed to infect humans.

HeV first emerged in September 1994 at a horse-training facility in Hendra, a suburb of Brisbane, Queensland, Australia. Multiple racehorses rapidly developed severe respiratory symptoms and high fever before succumbing to the disease. The trainer and one of his stable hands subsequently contracted the illness after contact with the affected horses; the trainer died from the infection (7). This outbreak was initially misidentified as equine influenza or glanders, but subsequent investigations confirmed the causative agent to be a previously unknown lethal pathogen. The virus subsequently caused outbreaks in multiple regions of Australia. As of February 2026, 105 equine cases had been reported in Australia. This broader total includes suspected or untested reports, whereas the annual dataset summarized in Table 1 comprises 90 laboratory-confirmed equine cases. The February 2026 figure refers to the cumulative number of reported equine cases available at that date, whereas the July 2025 dataset summarized in Table 1 includes 90 laboratory-confirmed equine cases; the July 2026 cut-off in Table 2 refers exclusively to the clinical-development status of vaccine candidates and antibody-based interventions. Seven human cases had been reported, including four deaths (8). HeV infection is associated with a high case fatality rate, estimated at approximately 80% in horses and 60% in humans (9).

**Table 1:** Summary of confirmed HeV cases in horses and corresponding locations in Australia, 1994–2025.

Year	Confirmed cases	Location	Year	Confirmed cases	Location
1994	9	Hendra, Brisbane; Mackay	2014	4	Gladstone; Murwillumbah (NSW); Beenleigh; Bundaberg
1999	1	Cairns	2015	4	Gympie; Lismore (NSW); Atherton Tableland; Murwillumbah (NSW)
2004	1	Townsville	2016	1	Casino (NSW)
2006	2	Murwillumbah (NSW); Peachester	2017	4	Lismore (NSW); Murwillumbah (NSW); Gold Coast Hinterland
2007	2	Cairns; Peachester	2018	1	Tweed Heads (NSW)
2008	8	Proserpine; Redlands	2019	1	Scone, Upper Hunter Valley (NSW)
2009	5	Bowen; Cawarral	2020	1	Murwillumbah (NSW)
2010	1	Tewantin	2021	1	West Wallsend near Newcastle (NSW)
2011	23	Beachmere; North Ballina; Gold Coast Hinterland; Mullumbimby; South Ballina; Ballina; Chinchilla; Boondall; Hervey Bay; Lismore; Kuranda; Macksville; Park Ridge; Wollongbar; Logan; Boonah; Beaudesert	2022	1	Mackay
2012	10	Ingham; Port Douglas; Cairns; Rockhampton; Mackay; Townsville	2023	1	Newcastle
2013	8	Kempsey (NSW); Macksville; Gold Coast Hinterland; Brisbane Valley; Atherton Tablelands; Mackay	2025	1	Southeast Queensland

Years with 0 confirmed cases are not listed; NSW = New South Wales. Suspected or untested reports were excluded from the total of 90 laboratory-confirmed cases.

**Table 2:** Clinical-stage *henipavirus* vaccine candidates and antibody-based interventions with potential relevance to HeV.

Candidate	Antigen or target	Current development stage as of July 2026	Relevance to Hendra virus	References
HeV-sG-V	Soluble HeV G glycoprotein	Phase I results published in 2025	The antigen is derived from HeV and can elicit NiV-cross-reactive neutralizing antibodies, supporting its potential development as a broadly protective *henipavirus* vaccine.	(92)
PHV02	G glycoprotein of the NiV Bangladesh strain; the vector additionally expresses Ebola virus glycoprotein (EBOV GP)	Phase I study NCT05178901 completed; Phase Ib study NCT06221813 completed.	PHV02 is primarily an NiV vaccine candidate; its relevance to HeV depends on evidence of cross-neutralization and cross-protection.	(93–95)
mRNA-1215	Chimeric prefusion F antigen linked to the G glycoprotein of the NiV Malaysia strain	Phase I results published in 2026	mRNA-1215 is an NiV vaccine candidate. Immune responses and cross-reactivity have been evaluated, but it is not an HeV-specific vaccine.	(97)
ChAdOx1 NipahB	G glycoprotein of the NiV Bangladesh strain	Phase IIa immunogenicity and safety study initiated in Bangladesh in December 2025; planned enrollment, 306 adults.	Preclinical protection was demonstrated in African green monkeys challenged with NiV. The Phase IIa Bangladesh study is registered as ISRCTN62461807. Its relevance to HeV requires direct experimental and clinical validation.	(98, 99)
m102.4	Receptor-binding G glycoprotein of HeV and NiV	Phase I safety study published; compassionate-use administration reported.	Cross-neutralizes HeV and NiV; protective in ferret and African green monkey models; investigational post-exposure prophylaxis.	(100, 103–105)

**Figure 1:**
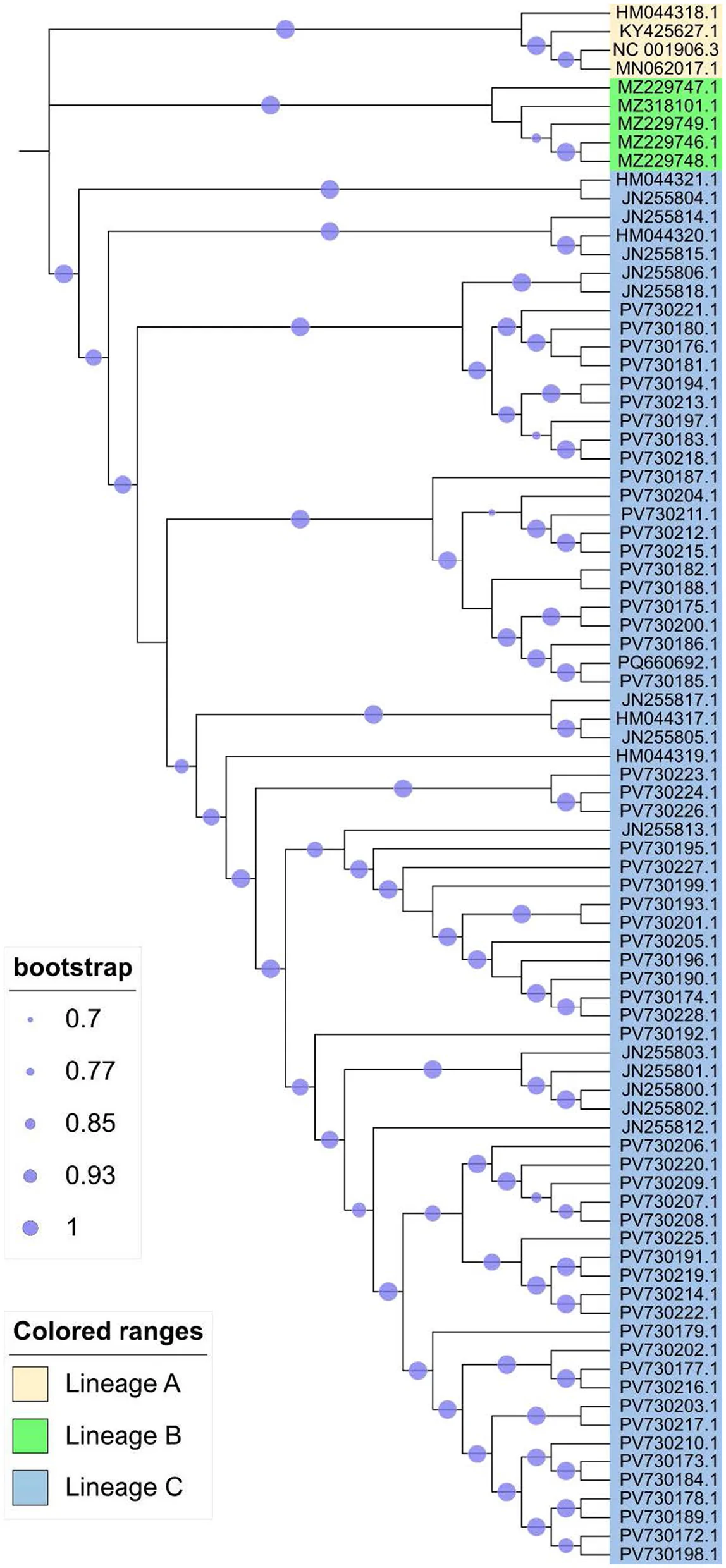
Maximum-likelihood phylogenetic tree of Hendra virus (HeV) based on complete and partial genome sequences. A total of 84 HeV nucleotide sequences were retrieved from the NCBI Nucleotide database, comprising 19 records annotated by GenBank as complete genomes and 65 records annotated as partial genomes. The complete-vs.-partial designation follows the GenBank annotation of each record rather than sequence length. The analyzed sequences ranged from 8,470 to 18,234 nt; no ambiguous-nucleotide-content exclusion threshold was applied. The shortest retained sequence was MZ229749.1 (8,470 nt), and the highest observed ambiguous-base proportion was 12.5% in PV730183.1. Five records carrying the NCBI UNVERIFIED flag (PV730199.1, PV730208.1, PV730209.1, PV730217.1, and PV730218.1) were retained because they originated from the same bat surveillance project and had sequence lengths close to the full HeV genome. The shorter and more ambiguous records contained sufficient homologous sequence for genome-scale comparison. The GenBank accession numbers and metadata for all 84 sequences are provided in Supplementary Table S1, which flags the retained records requiring clarification. Multiple-sequence alignment was performed using the ClustalW algorithm implemented in MEGA 12. The phylogenetic tree was inferred in MEGA 12 using the maximum-likelihood method under the Tamura–Nei nucleotide-substitution model, assuming uniform evolutionary rates among sites. Sites containing gaps or ambiguous nucleotides were retained, and gaps and ambiguous characters were treated as missing data rather than as observed nucleotide states. Branch support was evaluated using 1,000 bootstrap replicates, and support values are shown as proportions (0.70–1.00) at major nodes. The tree was midpoint-rooted, and Lineages A, B, and C are highlighted in yellow, green, and blue, respectively. The tree with the highest log likelihood (−47, 013.46) is shown. The final alignment comprised 84 nucleotide sequences and 18,276 aligned positions. Analyses were conducted in MEGA 12 (110–112). Because the dataset includes both complete and partial sequences and contains records with substantial ambiguous nucleotide content or NCBI “UNVERIFIED” status, the tree is presented as a descriptive summary of currently available HeV sequence diversity rather than as a formal evolutionary or phylodynamic analysis.

HeV comprises at least two genotypes: HeV genotype 1 (prototype) and genotype 2 (variant) (10). Both genotypes can spill over naturally into horses and cause severe, often fatal disease (11). The two genotypes cause similar clinical signs in infected horses and can both present as acute, severe disease. Whole-genome nucleotide identity between the two genotypes is approximately 83.5%, with slightly higher identity across coding regions (86.9%). Compared with prototype HeV, the variant has greater sequence variation in its noncoding regions, and its M-gene sequence contains multiple nucleotide mismatches. These mismatches can prevent conventional M-gene primers and probes from binding, leading to false-negative results in routine RT-PCR testing and contributing to the variant's initial nondetection (12). According to the phylogenetic analysis presented in Figure 1, although HeV genotype 1 (HeV-g1) is highly conserved, the currently available sequences can be grouped into two apparent sublineages, Lineages A and C. As a distinct genotype, HeV-g2 forms Lineage B, expanding the recognized sequence diversity of the virus. The genetic diversity of HeV is reflected not only in the divergence between HeV-g1 and HeV-g2, but also in variation among subclades within HeV-g1. This multilevel pattern may provide useful context for surveillance, although its implications for spillover risk should be interpreted cautiously. Research indicates that equine HeV infection stems from direct exposure to secretions from infected flying foxes (13). All infected humans had contact with bodily fluids from severely ill infected horses, or with equipment or environments contaminated by such fluids (14). Owing to their high pathogenicity, broad host range, and the absence of licensed human vaccines and specific therapeutics, HeV and NiV are important subjects of biosafety level 4 (BSL-4) research (15, 16).

Several published reviews have already integrated epidemiology, molecular virology, diagnostics, pathogenesis, vaccination, ecological drivers, and One Health control of HeV. Building on this literature, the present review provides a veterinary-oriented One Health synthesis with four specific emphases: the diagnostic consequences of HeV-g2; a structured comparison of the suitability and limitations of animal models; integration of equine vaccination with occupational-risk reduction; and an updated comparison of clinical-stage *henipavirus* vaccines and antibody-based interventions with potential relevance to HeV. The review therefore links fundamental virology with veterinary clinical evidence and practical prevention strategies without implying that earlier reviews were restricted to isolated topics.

## 2 The HeV genome and encoded proteins

HeV has multiple genotypes. Isolates from equine sources demonstrate high genetic similarity, with minimal nucleotide and amino acid variation, indicating extreme conservation. All the isolates shared an identical genome length of 18,234 nucleotides (nt), with whole-genome sequence variation less than 1% (17). This represents an increase of approximately 2,700 nt compared with other genera within the family *Paramyxoviridae* such as *Respirovirus* and *Morbillivirus*, constituting a key distinguishing feature from other paramyxoviruses. This larger genome is a distinguishing feature of the genus *Henipavirus* (15).

**Figure 2:**
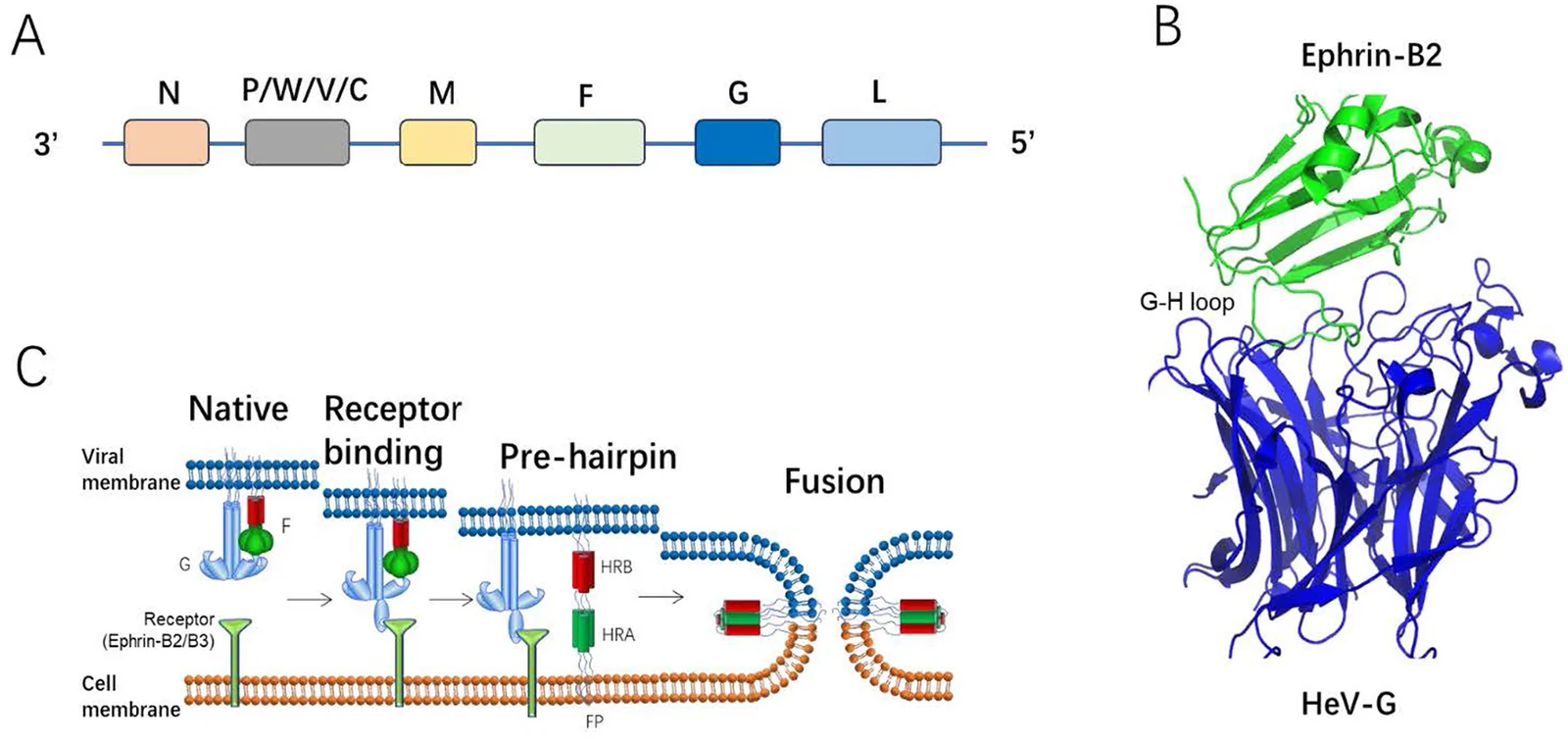
The HeV genome and its infection process. **(A)** HeV genome structure; **(B)** Crystal structure of the HeV G glycoprotein in complex with ephrin-B2 (PDB ID: 6PDL); **(C)** HeV fusion process with the target cell membrane.

The HeV genome encodes six structural proteins: the attachment glycoprotein (G), fusion protein (F), matrix protein (M), phosphoprotein (P), nucleoprotein (N), and polymerase protein (L) (18). The genome is similar to that of NiV, wherein the P gene undergoes RNA editing to express additional proteins—P, V, W, and C (Figure 2A). These proteins have been demonstrated to have the capacity to suppress antiviral immune responses, playing a crucial role in the virus's evasion of the host's innate immune defenses (19, 20). The G protein is a type II integral membrane protein capable of specifically recognizing and binding to the cell surface receptors Ephrin-B2 and Ephrin-B3, thereby mediating the attachment process between the virus and host cells. This step constitutes the critical initiation phase of viral entry into host cells (21). The F protein is initially synthesized as an inactive precursor polypeptide, F0. Following proteolytic cleavage, F1 and F2 are linked by disulfide bonds. The F protein becomes functional only after cleavage (22). The F protein requires co-participation with the G protein to function, forming a functional complex. When the G protein recognizes and binds to the host cell surface receptor Ephrin-B2 and Ephrin-B3, it initiates signal transduction, inducing a conformational change in the F protein and subsequently triggering the membrane fusion reaction (23). The M protein serves as the core component of viral assembly, primarily driving the formation and release of virus-like particles. As a key protein in viral particle production, it facilitates the efficient generation of infectious viral particles (24). Charge interactions between the α1 and α2 helices of the M protein are crucial for virus-like particle formation; a significant reduction in viral particle yield occurs after mutation (25). The interaction between the N and P proteins is pivotal for viral transcription and replication, which is achieved primarily through binding between the C-terminal disordered domain of the N protein and the C-terminal domain of the P protein (26). As a core functional protein for viral replication and transcription, the L protein has the largest molecular weight yet is the least abundant among viral proteins within the genus *Henipavirus* of the family *Paramyxoviridae*. Its central roles include viral genome replication, mRNA processing and modification, and the regulation of viral proliferation (27).

### 2.1 Key proteins involved in HeV infection

HeV initiates infection by binding its G glycoprotein to ephrin-B2 (or alternatively ephrin-B3) on host cell surfaces. Bonaparte et al. first demonstrated ephrin-B2 as the functional receptor (23); subsequent crystallographic analysis revealed extensive protein-protein interfaces between the β-propeller head of the G glycoprotein and the G-H loop of ephrin-B2, forming a stable complex (Figure 2B) (28). Ephrin-B2 is a type I transmembrane protein comprising approximately 330 amino acids and is highly conserved across vertebrates. Its high expression in the vascular endothelium, neurons, and respiratory epithelium explains the broad tissue tropism of the virus (29).

Unlike most paramyxoviruses, the HeV G glycoprotein lacks hemagglutinin or neuraminidase activity, and its receptor-binding head adopts a conserved six-bladed β-propeller structure that forms the principal domain for binding ephrin-B2 (15). Within the F protein, the F1 fragment is the key functional domain mediating fusion between the viral envelope and the host cell membrane. It contains a fusion peptide and two critical α-helical heptad repeat regions (HRA and HRB)—the N-terminal heptad repeat (HR) and the C-terminal HR—which can form a hairpin trimer structure (22). Research indicates that specific amino acid mutations in the NiV F protein, such as L104C/I114C, L172F, and S191P, stabilize the prefusion conformation. Identical mutations are equally effective in the HeV F protein, providing crucial insights for vaccine antigen design (30).

### 2.2 HeV infection process

HeV initiates infection by specifically binding to the ephrin-B2 receptor on host cell surfaces via its G glycoprotein, which induces a conformational change that transmits an activation signal to the F glycoprotein, prompting it to undergo a conformational rearrangement and assemble into a hairpin trimer whose generated mechanical force serves as the primary driving force for fusion between the viral envelope and the host cell membrane (23). Following activation, the HRA region of the F protein refolds and extends toward the target cell membrane, enabling the fusion peptide to insert into the lipid bilayer of the target cell membrane. Further rearrangement brings HRA (located near the N-terminus of the F protein) into proximity with heptad repeat B (HRB) (located near the C-terminus of the F protein), forming a stable six-helix bundle, which ultimately facilitates the complete formation of the fusion pore, accomplishing membrane fusion between the virus and the host cell, and the viral nucleocapsid is released into the cytoplasm, initiating the replication cycle (Figure 2C) (31, 32).

An understanding of the structural features and interactive mechanisms of HeV G and F proteins is foundational for translational development: mapping the receptor-binding epitopes on HeV G enables the rational design of subunit vaccines, vector vaccines, and broad-spectrum immunogens covering HeV-g2 variants, while clarifying the conformational changes of F protein triggered by G-receptor binding facilitates the screening and optimization of two classes of entry inhibitors—neutralizing monoclonal antibodies targeting HeV G to block receptor attachment, and small-molecule peptides that lock F protein in its pre-fusion state to suppress membrane fusion, laying a critical molecular basis for preventive equine and human vaccines and post-exposure therapeutic agents against HeV zoonotic spillover.

## 3 Epidemiological characteristics and transmission chain of HeV

### 3.1 Epidemiological characteristics

**Figure 3:**
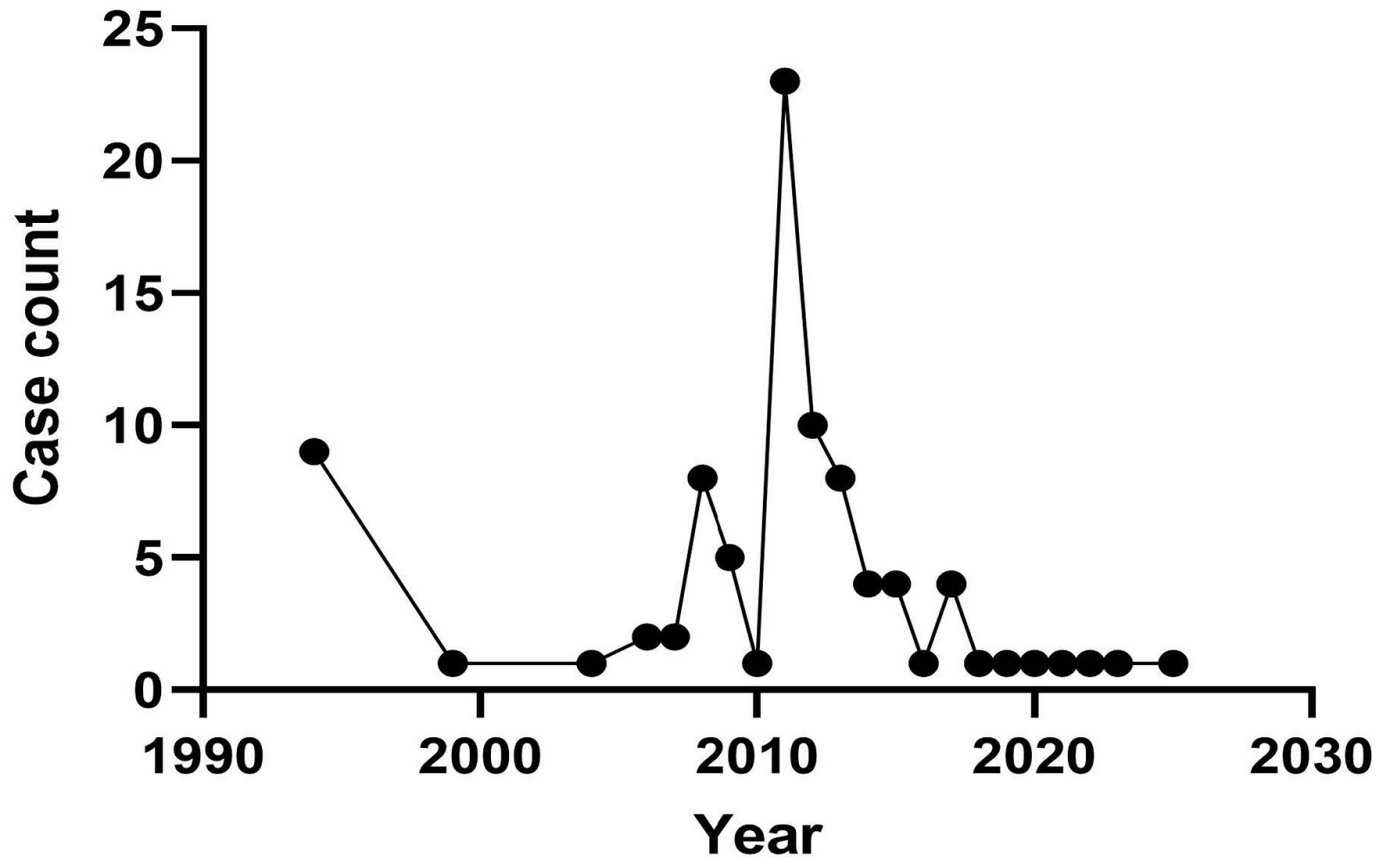
Temporal trend of annual confirmed HeV cases in horses, 1994–2025.

According to surveys, all confirmed HeV outbreaks and naturally acquired cases have been confined to Australia, with a distinct geographical concentration along the east coast. Most outbreaks have occurred in the coastal regions of Queensland and the northeastern corner of New South Wales; prior to 2014, the northernmost case was in Queensland, and the southernmost case was in Kempsey (New South Wales) (33, 34). In 2019, the southernmost case of HeV was detected in the Upper Hunter region of New South Wales (9), and by 2021, the virus had spread further south to the vicinity of Newcastle (35). This suggests that the range of the virus may expand southward and inland as the distribution of its fruit bat hosts shifts. The geographic distribution of HeV is closely linked to the habitats of fruit bats in the family *Pteropodidae*; hotspots for HeV infections in horses closely overlap with areas of high fruit bat density (36), and these bats are primarily found in the coastal and forested regions of eastern Australia (37). Cases of HeV in humans and horses are concentrated in areas where fruit bat habitats overlap with horse farms and human settlements, highlighting the role of dynamics at the human-wildlife interface in viral spillover (38, 39). Furthermore, the risk stems not only from local overlaps but also from how large-scale environmental changes shape bat behavior; these changes have altered the foraging dynamics of fruit bats, thereby driving contact events leading to virus spillover into horse populations (40). HeV spillover occurs sporadically, with events clustering during the winter months and being influenced by ecological and climatic factors (41, 42). Prior to 2010, HeV outbreaks in horses were sporadic; a total of 14 outbreaks were confirmed between 1994 and 2010. The unprecedented number of outbreaks reported in 2011 drew public attention and highlighted the unpredictability of HeV spillover. Since the virus was first identified, the overall incidence of HeV outbreaks has been low but persistent. According to data available as of July 2025, a total of 90 laboratory-confirmed cases of HeV infection in horses had been recorded in Australia (Table 1) (43). The low incidence rate may reflect the limited overlap among flying fox habitats, horse pastures, and human activity, together with the effects of the equine vaccination program introduced in Australia in 2012 and associated biosecurity measures (44). Following the introduction of the equine vaccine, the annual number of laboratory-confirmed equine cases declined overall. Vaccination is likely to have contributed to this trend by reducing susceptibility in horses and, consequently, the likelihood of occupational exposure in humans. However, the descriptive annual data do not establish vaccination as the sole cause of the decline. Other factors, including vaccine coverage, adherence to booster schedules, on-farm biosecurity practices, diagnostic awareness, flying fox ecology, and climatic conditions, may also have influenced the annual occurrence of spillover events (45). The annual trend is shown in Figure 3.

### 3.2 Transmission of HeV

**Figure 4:**
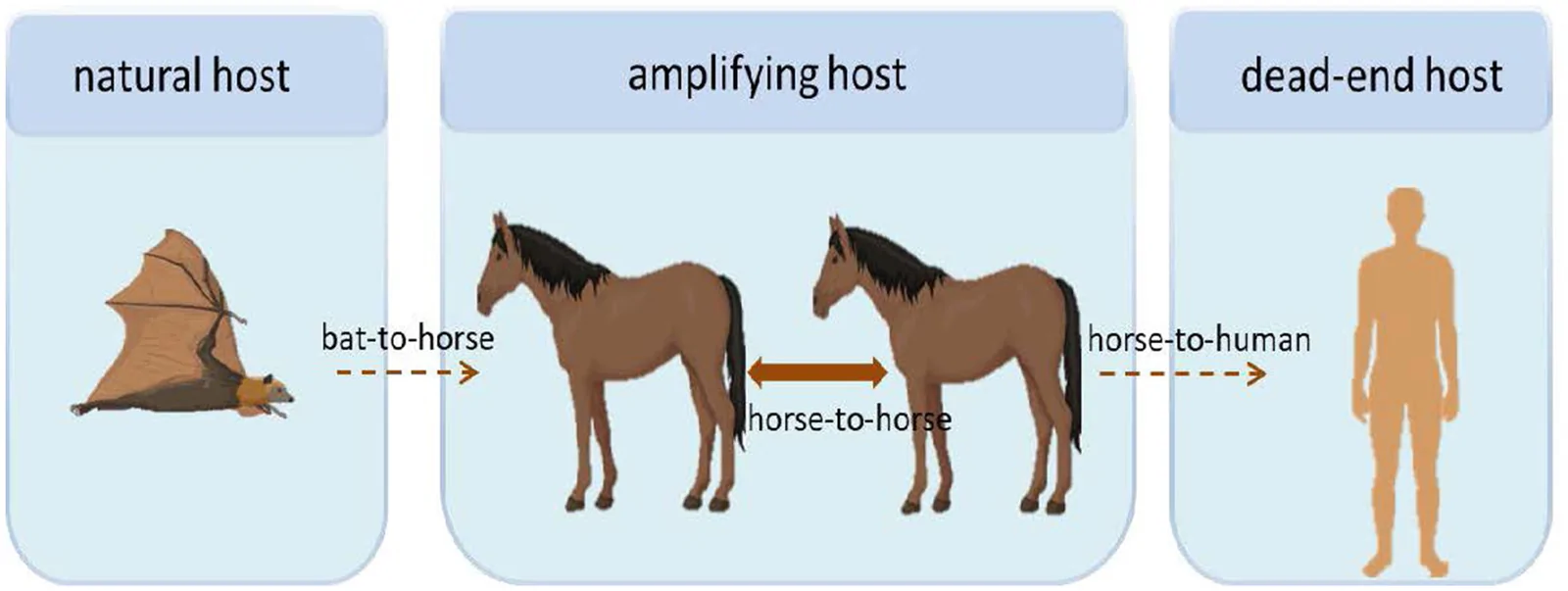
Transmission routes of HeV.

During the early stages of the outbreak, the source of infection remained unclear. Scientists therefore investigated wildlife potentially associated with the affected horse facility. Extensive serological investigations and viral isolation ultimately identified Australia's native flying foxes as the natural reservoir hosts of HeV (Figure 4) (46). Infected flying foxes typically exhibit no obvious clinical symptoms, enabling the virus to persistently circulate within their populations (47). On the basis of existing research evidence, equine infection is believed to occur through oral-nasal or conjunctival contact with virus-containing fruit bat urine. However, no experiments have directly confirmed the transmission mechanism from fruit bats to horses (48). Flying foxes are the natural reservoir hosts of HeV, whereas horses act as amplifying hosts and are currently the only mammalian species known to acquire infection directly from flying foxes (9). Infected horses can transmit the virus to other horses (48). Experimental HeV infection has also been demonstrated in dogs (49), pigs (50), hamsters (51), ferrets (52), cats, and guinea pigs (20). Human HeV infection occurs through close contact with bodily fluids or tissues from infected horses. All confirmed human cases involved a clear history of contact with sick horses, including during veterinary examination, care, postmortem procedures, or handling of infectious materials (53). There is currently no evidence that HeV can be transmitted between humans, nor are there any confirmed reports of direct transmission from fruit bats to humans. This limits its potential for large-scale human-to-human transmission, but also makes contact with infected horses the only documented and high-risk route of human infection. From the perspective of the complete bat–horse–human HeV transmission routes, establishing One Health surveillance systems is indispensable for effective HeV epidemic control. Equine vaccination acts as a core preventive intervention yet cannot contain viral spillover independently. Long-term ecological surveillance of flying fox populations can inform risk assessment and targeted prevention, but effective control requires an integrated combination of equine vaccination, on-farm biosecurity, occupational precautions, diagnostic preparedness, and ecological surveillance.

## 4 Clinical signs and symptoms

### 4.1 Symptoms in human infection

The total number of human HeV infections remains low. Clinical signs and symptoms typically emerge 5–21 days after exposure to HeV (54). Initial manifestations are predominantly nonspecific influenza-like symptoms, including fever, myalgia, headache, and fatigue (55). Severe cases may rapidly progress to respiratory failure, and interstitial pneumonia is a major pathological feature, particularly following close, direct exposure to infected horses or their bodily fluids and respiratory secretions (56). Owing to the limited number of human cases, the full clinical spectrum remains incompletely characterized. Neurological involvement may manifest as acute encephalitis or meningoencephalitis, with confusion, ataxia, or seizures. Postmortem examinations have frequently revealed multifocal necrosis in the brain parenchyma. Encephalitis may also occur as a delayed and often fatal complication (57, 58). Furthermore, HeV exhibits broad tissue tropism and causes systemic vasculopathy. Widespread vasculitis can affect microvascular endothelial cells in multiple organs, and histopathological findings include syncytium formation, thrombosis, inflammation, and ischemic necrosis, which may contribute to multiorgan dysfunction (59).

### 4.2 Clinical signs in infected horses

HeV causes severe, rapidly progressive disease in horses and often results in death within a short period. The incubation period is generally 3–15 days (60). During the subclinical phase of infection, viral genetic material is detectable via nasal swabs within just 2 days in experimental horses exposed to HeV through the oral and nasal routes, indicating that the respiratory epithelium is the primary site of initial viral entry (55). Early clinical signs may include reduced appetite, depression, high fever, and severe acute respiratory distress, with rapid progression to fatal interstitial pneumonia (20, 61). The principal clinical manifestations include fever, dyspnea, frothy nasal discharge, ataxia, myocarditis, and systemic vasculitis, with rapid deterioration over 2–3 days (62, 63). Neurological symptoms may occur in a minority of cases and are associated with cerebral vascular lesions, such as focal cerebral infarction or meningoencephalitis (64). The virus has been detected in nasal, oral, pharyngeal, and rectal secretions, as well as in urine (65).

### 4.3 Clinical manifestations in other animal species

Although horses usually develop overt and severe disease after HeV infection, experimentally infected animal species show variable clinical manifestations and disease severity.

#### 4.3.1 Pigs

Following oral or intranasal inoculation, infected pigs predominantly exhibit lethargy, respiratory signs, and fever. In some small-scale porcine models, neurological manifestations have also been observed (50).

#### 4.3.2 Cats

To date, no cases of natural HeV infection in cats have been documented. Following experimental inoculation, only a subset of the cats developed open-mouth breathing and dyspnea, while the remainder showed no overt clinical signs. Viral RNA was detected in renal tissue and urine (66).

#### 4.3.3 Non-human primates (African green monkeys)

Following intratracheal inoculation, the earliest clinical signs included reduced activity, piloerection, and mild lethargy. Disease progression was rapid, with subsequent manifestations including severe lethargy, marked impairment of mobility, and respiratory compromise. Pathological features included systemic vascular disease with pronounced involvement of the pulmonary and central nervous systems, closely resembling the disease observed in human cases (67, 68).

#### 4.3.4 Hamsters

Clinical outcomes are dose-dependent and influenced by age. Clinical signs typically appear within 24 h before death, with an acute, fulminant onset and no evident prodromal phase. The predominant pathological features are concurrent neurological and respiratory involvement accompanied by systemic viremia (51).

#### 4.3.5 Guinea pigs

Following HeV infection, guinea pigs may develop systemic vascular disease and pneumonia. Some individuals may progress to encephalitis; however, clinical signs are often mild and exhibit individual variability (69).

## 5 Diagnostic methods and differential diagnoses

Early and accurate diagnosis of HeV infection is essential for the timely implementation of animal isolation, infection control, contact tracing, and assessment of human exposure risk. However, the clinical manifestations of HeV infection in horses are highly variable and may include nonspecific fever, rapidly progressive respiratory disease, neurological abnormalities, colic-like signs, or sudden death. Because these clinical signs are nonspecific, definitive diagnosis must rely on laboratory testing (70).

Reverse transcription quantitative PCR (RT-qPCR) is the method of choice for confirming HeV infection (71). In suspected live horses, EDTA-anticoagulated whole blood, serum, and oral and nasal swabs may be collected. Depending on the clinical context, cerebrospinal fluid, urine, rectal swabs, and other respiratory samples may also be tested. In fatal cases, tissues such as lung, brain or spinal cord, kidney, spleen, tonsil, and lymph nodes may be collected. Conventional or semi-nested RT-PCR followed by nucleotide sequencing can be used for further confirmation and genotyping, although these methods are generally less sensitive than RT-qPCR. For cases with strong clinical or epidemiological suspicion, a single negative result cannot completely exclude infection. Repeat sampling should therefore be performed, and two or more assays targeting different genomic regions should be used (70).

Serological methods, including indirect ELISA, blocking ELISA, and IgM-capture ELISA, are mainly used for epidemiological surveillance, investigation of exposed animals, retrospective diagnosis, and assessment of immune responses. Because the currently available equine HeV vaccine is based on the HeV G glycoprotein, vaccination history must be considered when interpreting positive screening results. Antibody assays based on the G protein may detect antibodies induced by both vaccination and natural infection, whereas N-protein-based IgM assays and ELISA methods incorporating both G and N antigens may assist in distinguishing naturally infected animals from vaccinated animals (72–74).

The virus neutralization test remains the reference method for confirming HeV-specific neutralizing antibodies. Because HeV and NiV are closely related antigenically, a degree of cross-neutralization may occur. When differentiation is required, neutralizing antibodies against both viruses should be tested in parallel, and the results should be interpreted together with virus-specific molecular assays and epidemiological information (75).

Immunohistochemistry (IHC) can be used to detect viral antigens in formalin-fixed tissues collected from deceased animals. It also enables the spatial localization of viral antigens in relation to vascular endothelial injury, vasculitis, and other histopathological lesions, and is therefore primarily used for post-mortem confirmation and pathological assessment (70). Its limitations include the dependence of antigen preservation on fixation conditions and tissue quality, generally lower sensitivity than molecular assays, the requirement for well-validated virus-specific antibodies, and potential antigenic cross-reactivity among related henipaviruses.

Virus isolation may be performed using fresh, unfixed specimens, such as lung, brain, kidney, spleen, or other tissues expected to contain high viral loads, and can confirm the presence of replication-competent virus in susceptible cell cultures. This method is valuable for investigating newly emerging cases, resolving discordant diagnostic findings, and characterizing viral phenotypes and antigenic properties. However, it is time-consuming and cannot replace rapid RT-qPCR-based diagnosis. Given the substantial zoonotic risk associated with HeV, virus isolation and subsequent characterization must be conducted only in authorized high-containment laboratories by appropriately trained personnel. It should therefore not be considered a routine procedure in general diagnostic laboratories (70).

Because HeV infection lacks pathognomonic clinical features, it should be differentiated from other diseases that cause acute neurological or respiratory signs in horses (76). Neurological differential diagnoses mainly include equine *herpesvirus* 1 (EHV-1) myeloencephalopathy, Australian bat lyssavirus infection or rabies, arboviral encephalitis, tetanus, botulism, bacterial meningoencephalitis, and toxic, metabolic, or traumatic neurological disorders. Respiratory differential diagnoses mainly include equine influenza, EHV-1 or EHV-4 infection, equine viral arteritis, strangles, bacterial pneumonia or pleuropneumonia, and African horse sickness in areas with relevant epidemiological risk (77).

Given the close genetic and antigenic relationship between HeV and NiV, NiV infection should also be considered in the differential diagnosis. Both viruses can cause similar respiratory, neurological, vascular, and histopathological changes and may show cross-reactivity in some serological, immunofluorescence and immunohistochemical assays (78). Species-level differentiation should primarily rely on virus-specific RT-qPCR assays and nucleotide sequencing. When necessary, parallel or differential neutralization assays for HeV and NiV, together with epidemiological information, may be used for comprehensive interpretation (75, 79).

All currently available diagnostic methods have limitations, which may be compounded by sequence variation in HeV-g2. No single assay can reliably detect and differentiate all HeV variants. Future diagnostic development should prioritize mutation-tolerant, multitarget molecular assays that distinguish HeV-g1, HeV-g2, and NiV; serological antigens that differentiate vaccine-induced from infection-induced antibodies; and standardized criteria for interpreting combined test results. These improvements would enhance early detection and reduce missed diagnoses within a One Health surveillance framework.

## 6 Animal models

A mouse encephalitis model established by intranasal inoculation with HeV revealed focal encephalitis, activated microglia, and substantial accumulation of viral antigen at lesion sites. This model provides a practical platform for investigating HeV-induced neuroinflammation and testing interventions targeting viral immune evasion. However, it does not fully reproduce the systemic vasculitis and multisystem disease observed in naturally infected horses or humans (80).

Guinea pigs subcutaneously inoculated with a high dose of HeV developed vascular lesions in the majority of cases, and a minority developed microscopic encephalitic lesions. The virus was detectable in both vascular and neuronal tissues, confirming the suitability of this model for studying HeV encephalitis. Nevertheless, this model is less commonly used in contemporary vaccine or therapeutic evaluation than ferrets and nonhuman primates (69).

Following oral exposure to HeV, beagle dogs exhibited minimal clinical symptoms. However, the tonsils and lower respiratory tract serve as primary viral replication sites, with transmission to ferrets occurring via oral secretions. Studies of both natural and experimental infections have shown that dogs can support viral replication and transient shedding and may have limited potential for secondary transmission under conditions of close contact. However, there is currently no evidence that HeV can be maintained long term in dog populations or establish an independent transmission cycle (49).

Hamsters are highly susceptible to HeV infection and can show systemic viral dissemination, making them useful for pathogenesis studies and early-stage evaluation of passive antibody-based interventions. However, disease outcome in hamsters may be influenced by challenge conditions, and extrapolation to natural host disease should therefore be cautious (51, 81).

Cats reproduce several pathological features of experimental HeV disease, particularly pulmonary and vascular lesions, and therefore have historical value for comparative pathology and transmission-related studies. Nevertheless, their use in modern vaccine or therapeutic pipelines has largely been superseded by ferret and nonhuman primate models (82).

Ferrets are particularly useful for preclinical evaluation of vaccines and countermeasures, because clinical disease, productive infection, viral genome detection, and shedding-related endpoints can be assessed in this model (52).

African green monkeys represent the most clinically relevant model for human HeV disease and are particularly valuable for late-stage evaluation of vaccines and post-exposure therapeutics. However, their use is limited by cost, ethical considerations, small group sizes, and the requirement for high-containment facilities (68). Therefore, no single animal model fully captures all aspects of HeV infection; model selection should be guided by the specific research question, such as neuroinvasion, vascular pathology, transmission risk, vaccine efficacy, or therapeutic evaluation.

In summary, each HeV animal model has unique strengths and inherent drawbacks in recapitulating viral pathology, clinical signs and transmission traits (83, 84). Low-cost rodent models, including mice, hamsters, and guinea pigs, are useful for preliminary pathogenesis studies and antibody screening, but they do not consistently reproduce the complete multisystem disease observed in naturally infected horses or humans (84). Ferrets act as a well-balanced preclinical model for vaccines and therapeutics (85). African green monkeys are the most clinically relevant established model for late-stage evaluation of candidate vaccines and therapeutics, although their use is constrained by cost, ethical considerations, small group sizes, and high-containment requirements (68). Companion cats and dogs are only applicable to exploratory transmission studies. No single model can fully reproduce all features of HeV infection; researchers ought to select animals targeted to specific aims: neuroinvasion, vascular lesions, cross-species transmission, vaccine efficacy or post-exposure therapy assessment (85).

## 7 Vaccines and monoclonal antibody countermeasures

Vaccination is a fundamental approach to controlling viral infections. For HeV, multiple immunological strategies have been investigated, including monoclonal antibodies, viral vaccines, and recombinant viral-vector platforms (86). A central vaccine-development strategy uses the soluble HeV G protein (HeV-sG) as the primary antigen. This protein binds the host-cell receptors ephrin-B2 and ephrin-B3, induces neutralizing antibodies, and can block viral entry. The F protein mediates membrane fusion, and its prefusion conformation contains major neutralizing epitopes and is therefore an important determinant of protective efficacy (87, 88). Intramuscular administration of a soluble HeV G-protein subunit vaccine to adult African green monkeys induced high levels of G-specific IgG and neutralizing antibodies. Following viral challenge, vaccinated animals were completely protected, with no clinical signs, pathological lesions, or detectable viral replication (89). The same antigenic approach induced high-titer neutralizing antibodies and complete protection in experimental models including horses, ferrets, and African green monkeys. It was subsequently translated into a commercial equine vaccine and has informed human vaccine development (90). Deployment of the equine vaccine in Australia has provided effective protection in vaccinated horses and may reduce the risk of onward occupational exposure, illustrating the One Health value of interrupting the HeV transmission chain (91).

As of July 2026, several henipavirus-related human vaccine candidates based on different technological platforms had advanced into clinical development; however, no human vaccine had been licensed or approved. The recombinant soluble G-glycoprotein subunit vaccine HeV-sG-V completed a Phase I clinical trial, and published results showed a favorable safety and tolerability profile together with induction of NiV-specific antibody responses (92). PHV02, a recombinant vesicular stomatitis virus-vectored vaccine, has been evaluated in two registered early-phase studies: the Phase I study NCT05178901 and the Phase Ib prime-boost study NCT06221813 were completed (93–95). The lipid nanoparticle-formulated mRNA vaccine mRNA-1215 completed a Phase I first-in-human study and induced NiV-specific binding and neutralizing antibody responses; exploratory analyses indicated cross-reactive binding and neutralizing responses against HeV (96, 97). Overall, although these candidates have demonstrated preliminary safety and immunogenicity, additional later-phase clinical studies are required to evaluate protective efficacy and the durability of immune responses. ChAdOx1 NipahB is a replication-deficient chimpanzee adenovirus-vectored vaccine encoding the G glycoprotein of the Bangladesh strain of NiV. Preclinical studies demonstrated protection in African green monkeys challenged with NiV (98). The candidate is being evaluated in a Phase IIa immunogenicity and safety study in healthy adults in Bangladesh (99). Its potential relevance to HeV is based on antigenic relatedness within the genus *Henipavirus* and requires direct experimental and clinical validation.

Additionally, m102.4 is a potent, fully human neutralizing monoclonal antibody targeting the receptor-binding domain of the HeV and NiV attachment glycoprotein G. Its epitope overlaps the ephrin-B2/ephrin-B3 receptor-binding site, enabling m102.4 to prevent receptor engagement and block viral attachment and entry (100, 101). Post-exposure administration provided complete protection against lethal *henipavirus* disease in ferret and African green monkey models, including protection against HeV when treatment was initiated up to 72 h after exposure (102–104). On the basis of these findings, m102.4 has been administered on a compassionate-use basis as post-exposure prophylaxis following high-risk HeV or NiV exposure (Table 2). A randomized, placebo-controlled Phase I trial in healthy adults demonstrated an acceptable preliminary safety and pharmacokinetic profile (105). However, the study was not designed to evaluate efficacy in infected patients, and the protective efficacy, optimal dosing regimen, and therapeutic window of m102.4 have not been established in controlled human studies. Therefore, m102.4 remains an investigational countermeasure rather than an approved treatment.

The licensed equine HeV subunit vaccine effectively reduces the risk of viral spillover and supports the maintenance of herd immunity in horses within a One Health framework. However, its protective efficacy depends on regular booster immunization, and it is not approved for human prophylactic use. Emerging human vaccine platforms offer the potential to induce cross-protective immunity against both HeV and NiV while enabling flexible and efficient vaccine development. Nevertheless, all current candidates remain at early stages of clinical development, and definitive evidence regarding protective efficacy and long-term immune durability is still lacking, thereby precluding formal licensure. In addition, the protective efficacy of the monoclonal antibody m102.4 in humans and its optimal dosing regimen have not yet been established, while high manufacturing costs and stringent cold-chain requirements may substantially limit its clinical scalability. Collectively, no single vaccine or antibody-based intervention currently provides comprehensive prevention and control of HeV infection. Large-scale clinical validation, comparative assessment across technological platforms, and optimization of standardized intervention protocols are therefore required to establish an integrated One Health strategy tailored to the zoonotic transmission characteristics of HeV.

## 8 New challenges to public health

In January 2026, an outbreak of NiV occurred in West Bengal, India, prompting neighboring countries such as Thailand and Nepal to increase vigilance and strengthen health screening for travelers arriving from India. This outbreak once again underscored the severe threat posed to global public health by the genus *Henipavirus*, which is a group of highly lethal zoonotic pathogens. In 2021, a new genotype of HeV, HeV-g2, was identified in Australia. Whole-genome sequencing showed that it shared high nucleotide homology with the prototype strain. The spillover distribution of this variant has extended southward to the central coast of New South Wales and southeastern Queensland, suggesting an expanded geographic range of potential HeV spillover risk (35). In addition, sequence differences between HeV-g2 and HeV-g1 in some molecular diagnostic target regions may limit the ability of PCR and RT-qPCR assays primarily developed for HeV-g1 to fully detect HeV-g2 (12, 106). The discovery of HeV-g2 therefore highlights the need to incorporate assays capable of detecting multiple HeV genotypes, or HeV-g2-specific assays, into future surveillance and diagnostic programs to reduce missed detection and improve early identification.

Virus-positive samples have been detected frequently in the Queensland–New South Wales border region, with a more pronounced pattern during the winter months (45). Increased viral shedding may expand the spatial distribution of spillover risk and increase the number of potential outbreak sites (107).

Evidence from Southeast Asian bats indicates substantial regional diversity of related *henipaviruses* and a possible broader *henipavirus* spillover risk. Serological and molecular findings from bats in Indonesia and neighboring areas do not demonstrate circulation or transmission of HeV in Southeast Asia (108, 109). Continued regional surveillance should therefore distinguish HeV-specific evidence from evidence relating to other or unclassified *henipaviruses*.

## 9 Conclusions and outlook

Collectively, HeV should be viewed as a veterinary and One Health threat in which horses represent both the principal clinically affected domestic species and the critical intervention point between flying fox reservoirs and humans. The emergence of HeV-g2 has broadened the recognized geographic and genetic diversity of the virus and highlights the need for genotype-inclusive molecular assays, vaccination-history-aware serological interpretation, and sustained surveillance at the wildlife–livestock interface. Animal models should be selected according to the research question: small-animal and ferret models are valuable for mechanistic and early countermeasure studies, whereas African green monkeys most closely reproduce the severe multisystem vascular, respiratory, and neurological disease observed in humans.

Countermeasure development illustrates a translational continuum from the licensed equine G-glycoprotein vaccine to human *henipavirus* vaccine candidates and monoclonal antibodies. Several soluble glycoprotein, viral-vector, and mRNA candidates have completed Phase I evaluation, and an adenoviral-vectored Nipah vaccine candidate has entered Phase II; nevertheless, no human vaccine or HeV-specific therapeutic has been licensed.

This review provides a veterinary-oriented One Health synthesis of HeV biology, transmission, clinical disease, diagnostics, animal models, equine vaccination, and clinical-stage countermeasures. Its specific contributions are the emphasis on HeV-g2-associated diagnostic limitations, structured comparison of animal-model suitability, integration of equine vaccination with occupational-risk reduction, and updated comparison of vaccine and antibody candidates potentially relevant to HeV. Current evidence supports combined prevention based on equine vaccination, farm biosecurity, occupational precautions, diagnostic preparedness, and ecological surveillance. Further work is needed to improve genotype-inclusive diagnostics, define correlates and durability of protection, and establish the efficacy of investigational human vaccines and antibody-based interventions.
